# Eph-ephrin signaling and its potential role in female reproductive tract development

**DOI:** 10.1007/s11033-025-11154-2

**Published:** 2025-10-16

**Authors:** Pankaj Prasun

**Affiliations:** Division of Genetics, Department of Pediatrics, American Family Children´s Hospital, University of Wisconsin-Madison, 1500 Highland Ave Madison, Madison, WI 53705 USA

**Keywords:** Female reproductive system, Uterine malformation, Müllerian duct, EFNB1, Craniofrontonasal syndrome, Eph-ephrin, Ephrin

## Abstract

Eph-ephrin signaling plays crucial role in the development of nervous and cardiovascular system. Its role in female reproductive system development is unknown. Clinical observations in a rare developmental disorder, Craniofrontonasal syndrome, underscores the potential role of Eph-ephrin signaling in the development of female reproductive tract. In this minireview, the basics of Eph-ephrin signaling and its known role in development have been discussed. Putative role of Eph-ephrin in female reproductive tract development is discussed based on the available clinical evidence and animal studies.

## Introduction

Eph (erythropoietin- producing human hepatocellular carcinoma cell) receptors are a family of cell-surface receptors which bind to ephrin (Eph family receptor-interacting protein) ligands on the neighboring cells. The Eph-ephrin complex coordinates contact based bidirectional cellular communication and plays crucial role in developmental processes by primarily regulating cell repulsion and adhesion [1]. Eph-ephrin signaling plays vital role in cell migration, tissue boundary determination, and tissue segmentation during development [2, 3]. Its role is extensively studied in the development of nervous system specifically axonal guidance, synaptogenesis, and hind brain segmentation [4–6]. In addition, Eph-ephrin signaling is important for topographic map generation in brain such as the retinotectal mapping [7]. Apart from the nervous system, Eph-ephrin signaling is also implicated in angiogenesis and vascular remodeling during cardiovascular development [8–10]. Given its role in the very basic developmental processes such as tissue patterning and differentiation, Eph-ephrin system is considered crucial for the development of all organ systems [2, 11]. However, there is very limited information on its role in the development of the female reproductive system. In this mini review, the potential role of Eph-ephrin signaling in female reproductive tract development is discussed based on the available clinical and laboratory evidences.

## Structure of Eph-ephrin complex

**Fig. 1:**
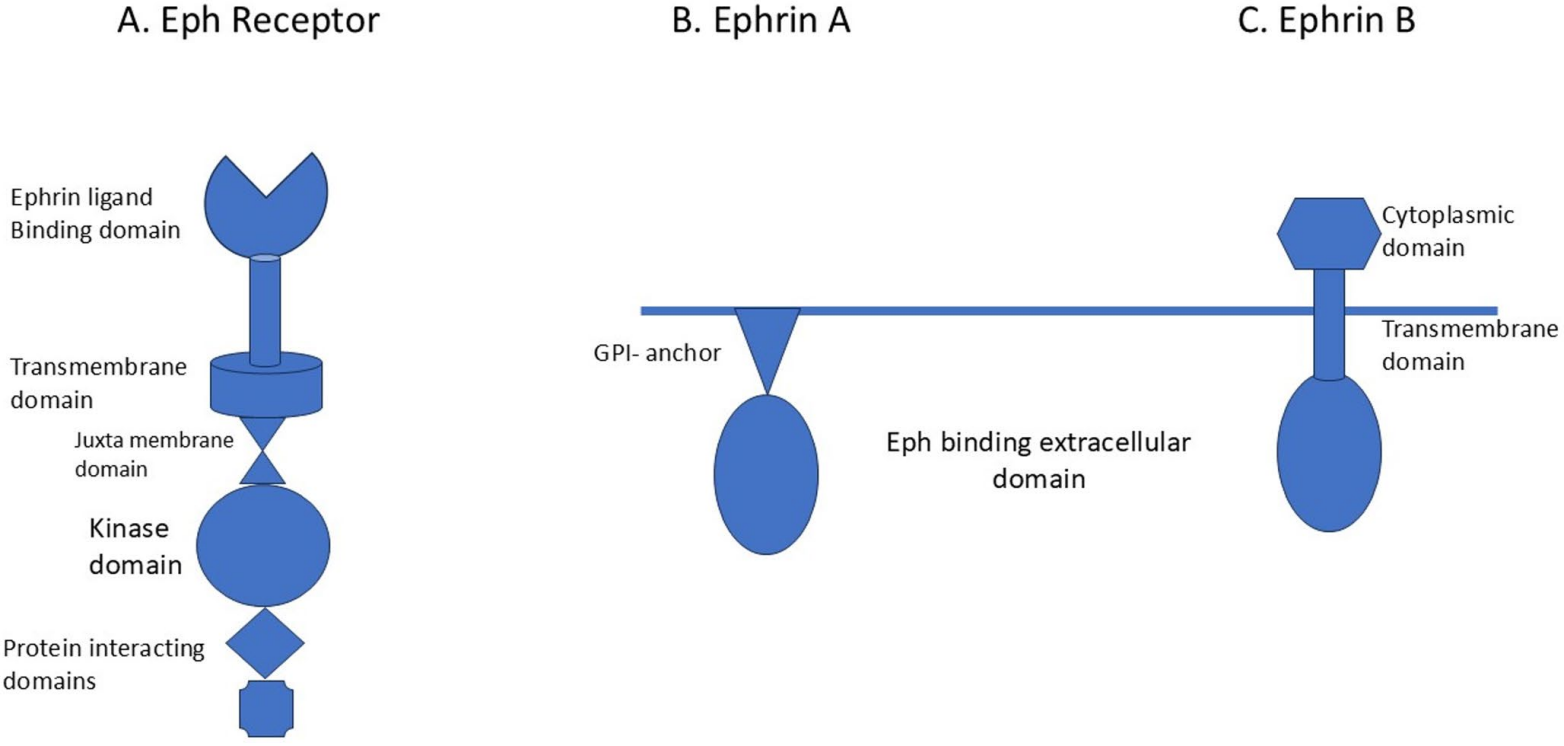
The simplified structure of an Eph receptor, and ephrin ligands

There are 2 classes of Eph and ephrin – A and B, and each class has several subtypes. There are 9 subtypes of Eph A and 5 subtypes of Eph B receptors in mammals [12]. All Eph receptors share the similar structure: a N- terminal extracellular domain through which it interacts with ephrin ligands followed by a short transmembrane domain, a juxta membrane region, a large kinase domain, and 2 domains for protein interaction (Fig. 1A) [13]. There are 5 subtypes of ephrin A and 3 subtypes of ephrin B [12]. Ephrin As have an extracellular receptor binding domain which is anchored to cell membrane by glycosylphosphatidylinositol (GPI) anchor (Fig. 1B). Ephrin Bs have an extracellular receptor binding domain followed by transmembrane domain and a small cytoplasmic domain (Fig. 1C). Eph receptors and ephrin ligands interact promiscuously within the subclass [11]. Eph As prefer ephrin As while Eph Bs prefer ephrin Bs with some exception [12].

GPI: glycosylphosphatidylinositol.

### Eph-ephrin signaling

**Fig. 2:**
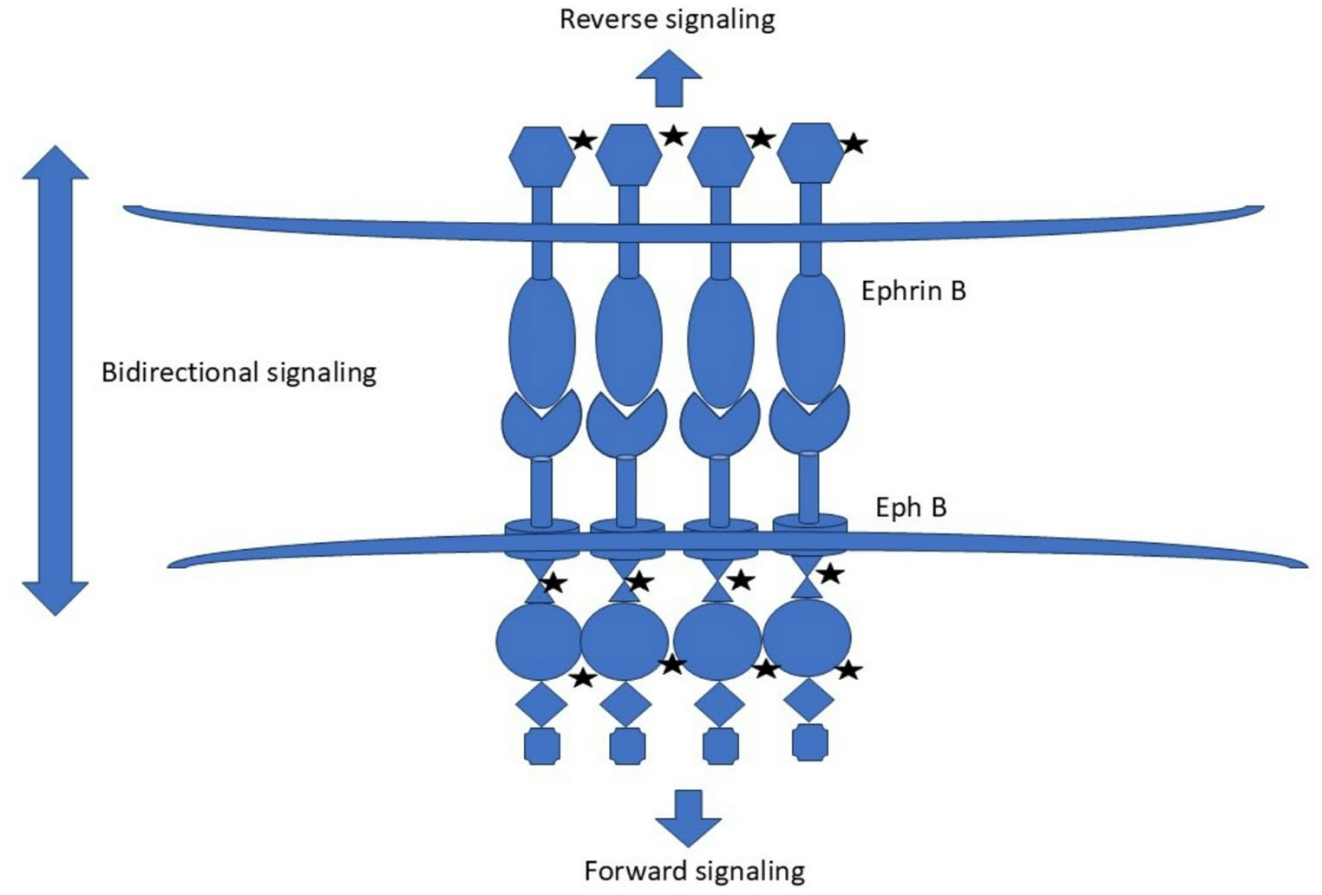
Bidirectional signaling from Eph-ephrin complex (the stars symbolize phosphorylation)

Eph-ephrin signaling is contact dependent. Following cell-cell contact, Eph receptor and ephrin ligand bind with high affinity. This leads to membrane clustering of Eph-ephrin complexes in an oligomeric structure. This is followed by autophosphorylation of conserved tyrosine residues in the juxta membrane region and disinhibition of the kinase domain of the Eph receptor [14]. This leads to phosphorylation of the downstream targets and propagation of a signal cascade, the end result of which usually is alteration of cellular cytoskeleton causing either cellular migration or adhesion depending on the context [15]. This cascade of event in Eph receptor bearing cell is called “forward signaling”. However, Eph-ephrin system can also induce signaling in the ephrin ligand bearing cell by phosphorylation of the cytosolic domain and subsequent activation of downstream pathways. This is called “reverse signaling” [16]. Bidirectional signaling from Eph-ephrin system is utilized during developmental processes such as tissue segregation and boundary formation (Fig. 2) [2, 14, 16].

## Overview of female reproductive tract development

Gonadal development is sex chromosome dependent [17]. In early embryonic stage, both male and female have paired Wolffian (mesonephric) and Müllerian (paramesonephric) ducts which serve as the precursors of male and female reproductive tracts, respectively. In embryos with XY chromosome, the Müllerian ducts regress due to anti-Müllerian hormone (AMH) produced by the embryonal testes while testosterone induces differentiation of Wolffian ducts into the male reproductive tract. In embryos with XX chromosome, the Wolffian ducts regress due to absence of testosterone while the absence of AMH leads to stabilization and differentiation of the Müllerian ducts into fallopian tubes, uterus, and the upper third of vagina [18, 19].

**Fig. 3:**
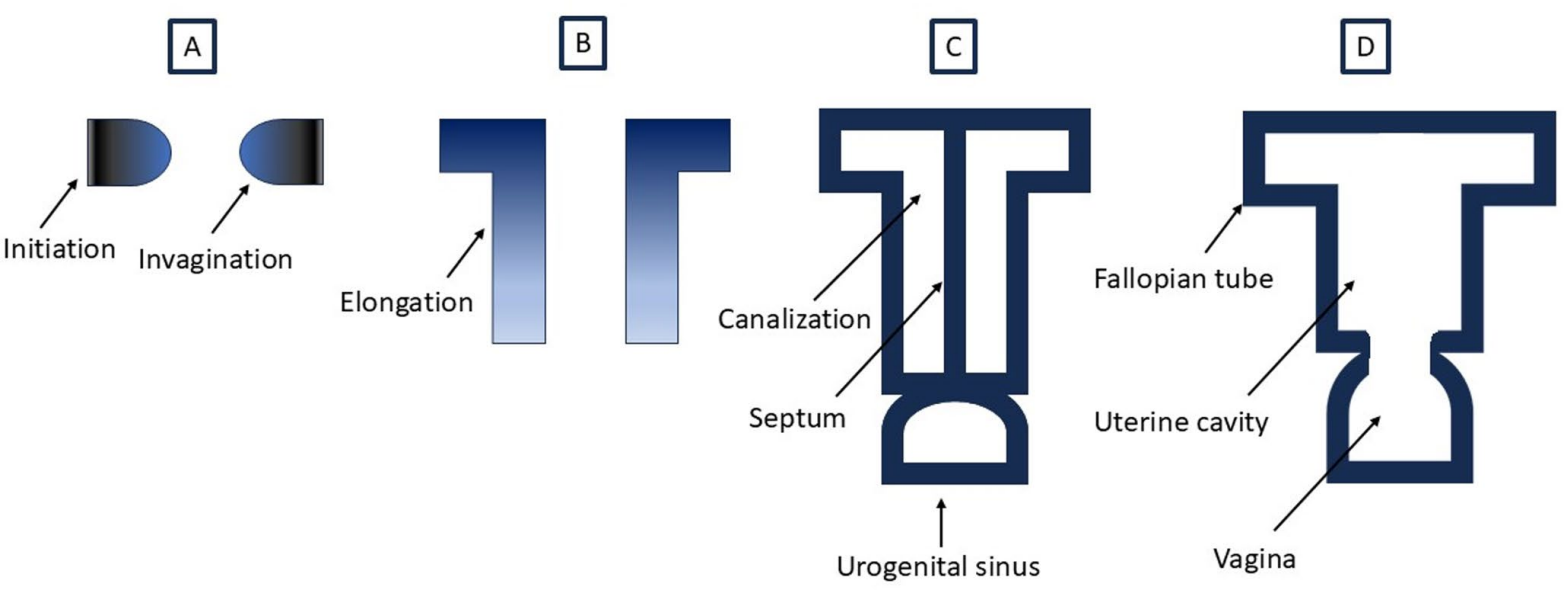
The simplified outline of female reproductive tract development. The placode-like thickening at coelomic epithelium marks Müllerian duct initiation which is followed by invagination of the specified cells (**A**), its caudal elongation (**B**), canalization, midline fusion, and septum formation (**C**). Resorption of the septum leads to uterine cavity formation (**D**). Caudal end of the fused Müllerian ducts forms the upper third of vagina

There are three phases in the development of Müllerian duct – initiation, invagination, and elongation [20]. Initiation involves placode-like thickening of the coelomic epithelium from where Müllerian duct specified cells invaginate laterally and caudally. The Müllerian ducts then elongate caudally and fuse in midline to form a T -shaped structure. These phases are completed by the week 11 of gestation. The canalization of the Müllerian ducts results in two lumens separated by a septum in the caudal portion. Resorption of septum results in formation of the uterine cavity (Fig. 3). The caudal end of the “T” fuses with urogenital sinus to form the upper third of vagina. The development of female reproductive tract is generally complete by the week 20 of gestation.

The initiation, invagination, elongation, canalization, and septal resorption of the Müllerian ducts are intricate processes [18, 20–22]. Molecular mechanisms underlying these processes are incompletely understood. However, it is likely that Eph-ephrin signaling is involved in each phase of the Müllerian duct development. The initiation phase requires fate determination and differentiation of a group of coelomic epithelial cells into Müllerian duct precursor cells. Eph-ephrin signaling is known to be associated with fate determination and differentiation during early embryonal morphogenesis in different tissues [23]. The invagination and elongation phases require coordinated and guided cell migration. Cellular migration and guidance are the key functions of Eph-ephrin system. This role of Eph-ephrin signaling is well established in axonal guidance, neural crest migration, and neuronal migration over long distances [24]. There is some evidence that Eph-ephrin signaling may have role in the Müllerian duct canalization and septal resorption as well. In approximately 18% of EphA1 null female mice, imperforate uterovaginal development leading to hydrometrocolpos was seen [25]. It was postulated that EphA1 is involved in hormone induced apoptosis during female reproductive tract canalization. Eph-ephrin signaling in female reproductive tract development is currently unrecognized but provides intriguing prospects for further research in female reproductive embryology.

### Molecular genetics of female reproductive tract development

Eph-ephrin signaling plays critical role in embryonal development. As mentioned above, it is also likely to be involved in several aspects of female reproductive tract development. The expression pattern of Eph-ephrin complexes during development is highly specific, graded, and overlapping/complementary [1, 11]. They act as effector molecules which expression is under transcriptional and post transcriptional control [26]. Transcription factors have emerged as the main regulators of Eph receptor expression [27]. Much of the current understanding of the molecular genetic mechanisms of female reproductive tract development comes from animal studies. Expression of the transcription factor Lim1 is crucial for fate determination of the coelomic epithelial cells for the initiation of Müllerian duct [28]. The transcription factor Wnt4 is required for invagination and elongation of the Müllerian duct [29]. Pax2, another transcription factor, is also needed for the elongation phase [30]. Following Müllerian duct formation, its differentiation in the anteroposterior and radial axis leads to formation of fallopian tubes, uterus, and upper vagina. This process involves interaction with mesenchyme and graded expression of the Hoxa family of the transcription factors (Hoxa9, Hoxa10, Hoxa11, Hoxa13) [22, 31]. Apart from the Hoxa family of transcription factors, Wnt transcription factors also regulate Müllerian duct differentiation [32]. Diethylstilbestrol (DES) is a synthetic estrogen which was previously used to prevent miscarriage. In utero exposure of diethylstilbestrol (DES) leads to Müllerian duct abnormalities. Gene expression studies have shown altered activity of Hox, Wnt, and Eph family of genes in Müllerian ducts of mice female fetuses exposed to DES in-utero [33, 34].

### Müllerian duct anomalies and genetic syndromes

Müllerian duct anomalies arise from the abnormal development of Müllerian duct. They are relatively common with an incidence of about 5.5% in the general population [35]. There are several types of Müllerian duct anomalies. Uterine agenesis/hypoplasia is caused by agenesis or underdevelopment of the Müllerian ducts. Unicornuate uterus is caused by the arrest of the development of one of the Müllerian ducts. Uterus didelphys is caused by the failure of fusion of the Müllerian ducts. Bicornuate uterus results from incomplete fusion of the Müllerian ducts. Septate uterus is the most common Müllerian duct anomaly and results from defective resorption of the septum between Müllerian ducts. Transverse vaginal septum results from defective resorption of the septum between urogenital sinus and caudal fused end of the Müllerian ducts (Fig. 3).

Genetic syndromes in humans associated with female reproductive tract anomalies provide further insight into the molecular mechanisms of female reproductive tract development [36]. Hand -foot-genital syndrome is caused by autosomal dominant pathogenic variation in *HOXA13*. It is characterized by hand defects, urinary tract anomalies, and Müllerian duct anomalies [37]. Mayer-Rokitansky-Kuster-Hauser (MRKH) syndrome is characterized by Müllerian agenesis where fallopian tubes, uterus, and upper vagina are absent. External genitalia and lower third of vagina are typically normal. MRKH syndrome may present with isolated Müllerian agenesis or associated with malformations in other organ systems. MRKH syndrome is usually sporadic. However, familial aggregation has been described suggesting a genetic etiology [38]. Pathogenic variations in *WNT4*,* TCF2*,* LHX1* (also known as *LIM1*) and *WNT7A* are associated with MRKH syndrome [36, 38, 39]. In addition, chromosome deletions affecting *TCF2*,* TBX6*, and *LHX1* are also associated with MRKH syndrome [38, 40].

The genes associated with the above-mentioned syndromes regulate transcription, and hence it is very likely that they mediate their actions by modulating Eph-ephrin signaling as mentioned in the previous section. For example, EphB receptor genes are targeted by Wnt signaling [41]. Similarly, Hox transcription factors have emerged as the key transcriptional regulators of the Eph receptor genes [26]. Perhaps the most convincing evidence of association of Müllerian duct anomalies with Eph-ephrin signaling is demonstrated in Craniofrontonasal syndrome caused by pathogenic variations in *EFNB1* encoding ephrin B1.

### Craniofrontonasal syndrome: linking Eph-ephrin signaling to female reproductive tract development

**Fig. 4:**
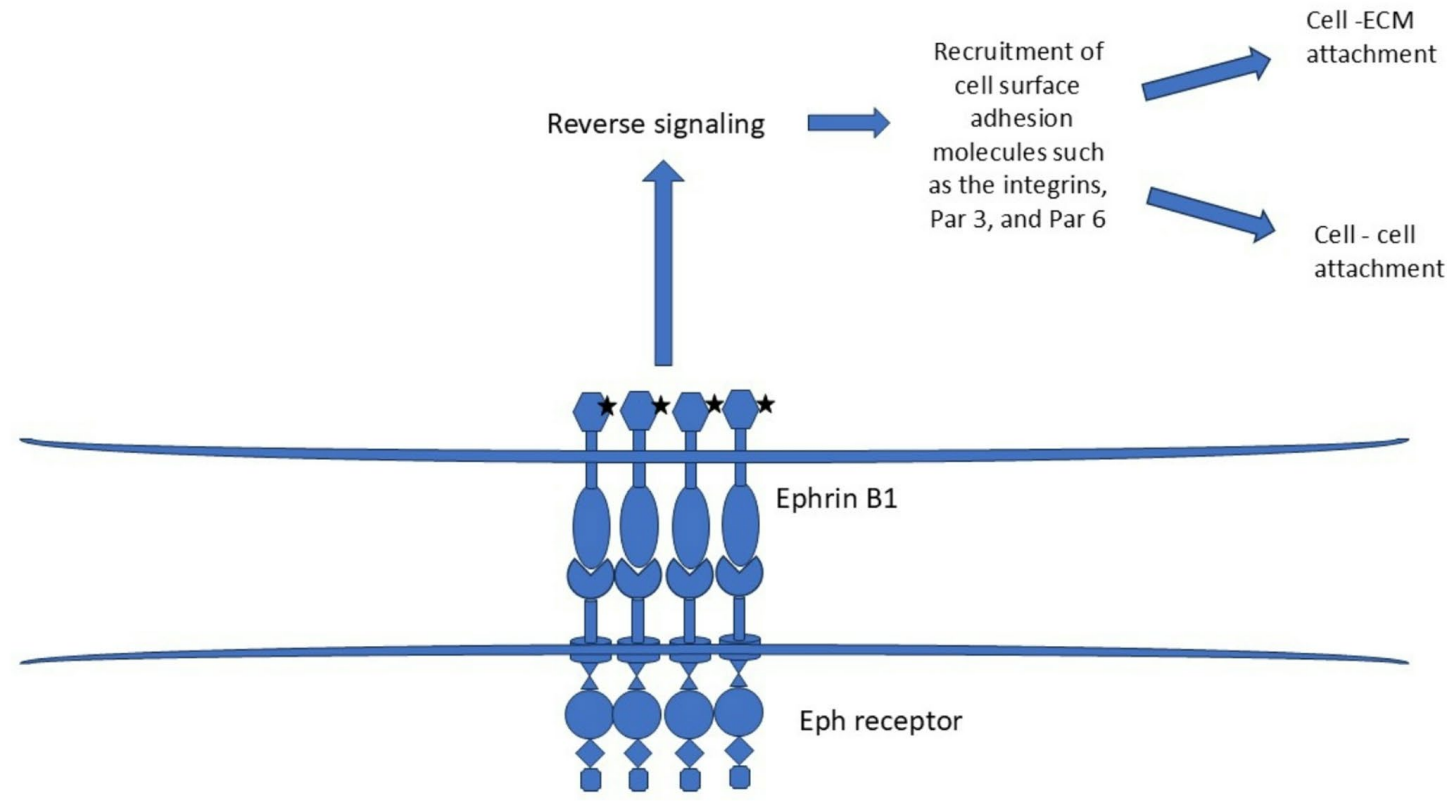
Postulated mechanism of Müllerian duct fusion abnormality in Craniofrontonasal syndrome. Ephrin B1 upon activation by phosphorylation recruits cell adhesion molecules such as the integrins, Par 3, and Par 6 which mediate cell- cell adhesion and cell – extracellular (ECM) attachment required for the Müllerian duct fusion

Craniofrontonasal syndrome (CFNS) (OMIM: 304110) is a rare developmental disorder characterized by coronal craniosynostosis, wide set eyes, midline nasal groove, cutaneous syndactyly, and duplication of the first digit [42]. In addition, agenesis of corpus callosum, cleft lip/palate, sloping shoulders, and low set breasts are commonly associated. Midline uterine malformations such as bicornuate uterus, uterus didelphys, and septate uterus have been described with CFNS [43–45]. In one patient didelphys uterus was found with septate vagina [43]. At our genetics center, we are following an individual with CFNS who has septate uterus in addition to the other characteristic features of this condition. CFNS is caused by pathogenic variations in *EFNB1* which encodes for ephrin B1. Eph B- ephrin B signaling regulate various developmental processes such as cell migration and adhesion as explained above [3]. Ephrin B1 modulates cellular adhesion by controlling tight junction formation by recruiting adhesion molecules such as integrins, Par 3, and Par 6 at the cell surface [46, 47]. It plays a significant role in neural tube closure during early development. This is mediated by adhesion of the apical progenitor cells [48]. *EfnB1* deficient mice embryo demonstrated morphological abnormalities similar to neural tube defects [48]. A similar mechanism for Müllerian tube anomalies in CFNS can be postulated (Fig. 4).

*EFNB1* pathogenic variants probably results in Müllerian duct anomalies by impairing midline fusion of the Müllerian ducts. The didelphys and bicornuate uterus seen in CFNS result from varying degree of incomplete fusion of the Müllerian ducts. Presence of didelphys uterus with longitudinal vaginal septum in one patient implicates complete non-fusion of the Müllerian ducts [43]. Septate uterus is the due to defective resorption of the septum formed by Müllerian duct fusion. Eph-ephrin signaling is also implicated in apoptosis needed for the septal resorption and formation of the uterine cavity during development [25].

CFNS is inherited in X-linked dominant manner. Females with this condition have more severe manifestation compared to males, which is interesting since males have only one copy of *EFNB1.* This is explained by the cellular interference phenomenon [49, 50]. In female, one of the X-chromosomes undergoes random inactivation. This results in mosaicism at the tissue level as there is a mixed population of cells where either the X-chromosome harboring the normal copy or the defective copy of *EFNB1* is active. This results in more severe disruption of intercellular signaling compared to males where the cell population in terms of ephrin B1 activity is uniform and likely compensated by other Eph-ephrin complexes due to promiscuity of Eph-ephrin binding [11, 51].

### Concluding remarks

The role of Eph-ephrin signaling in nervous system and cardiovascular system development is well recognized. Due to its vital role in contact based intercellular communication, it is likely involved in early development of other organ systems as well. However, the role of Eph-ephrin in female reproductive system development is unrecognized at this time. The presence of midline uterine and vaginal structural defects in CFNS underscores its role in female reproductive tract development.

There is emerging evidence that intercellular communications facilitated by the Eph-ephrin system are not only utilized in the developmental processes, but also coordinate diverse physiological processes and homeostasis in different organ systems [52–55]. Dysregulation of this system is implicated in cardiovascular, neurological, and retinal diseases [10, 56–58]. Its role in cancer development and progression is particularly intriguing [12]. In female reproductive system, proper functioning of this complex is thought to modulate folliculogenesis, ovulation, embryo transport, implantation, and placentation, while its dysregulation has been linked with pathologies such as polycystic ovarian disease, ectopic pregnancy, ovarian cancer, and endometrial cancer [59]. Role of Eph-ephrin signaling in the development of female reproductive tract should be explored further. It will not only help our understanding of the molecular pathogenesis of the developmental anomalies but will also enhance understanding of the female reproductive tract pathologies, ultimately opening the avenues for molecular and gene-based therapy [60].

## Data Availability

No datasets were generated or analysed during the current study.
